# Launching a Novel Preclinical Infrastructure: Comparative Oncology Trials Consortium Directed Therapeutic Targeting of TNFα to Cancer Vasculature

**DOI:** 10.1371/journal.pone.0004972

**Published:** 2009-03-30

**Authors:** Melissa C. Paoloni, Anita Tandle, Christina Mazcko, Engy Hanna, Stefan Kachala, Amy LeBlanc, Shelley Newman, David Vail, Carolyn Henry, Douglas Thamm, Karin Sorenmo, Amin Hajitou, Renata Pasqualini, Wadih Arap, Chand Khanna, Steven K. Libutti

**Affiliations:** 1 Comparative Oncology Program, Center for Cancer Research, National Cancer Institute, Bethesda, Maryland, United States of America; 2 Surgery Branch, Center for Cancer Research, National Cancer Institute, Bethesda, Maryland, United States of America; 3 University of Tennessee, Knoxville, Tennessee, United States of America; 4 University of Wisconsin, Madison, Wisconsin, United States of America; 5 University of Missouri, Columbia, Missouri, United States of America; 6 Colorado State University, Ft. Collins, Colorado, United States of America; 7 University of Pennsylvania, Philadelphia, Pennsylvania, United States of America; 8 Department of Gene Therapy and Division of Medicine, Imperial College London, Wright-Flemming Institute, London, United Kingdom; 9 David H. Koch Center, The University of Texas M. D. Anderson Cancer Center, Houston, Texas, United States of America; University of Florida, United States of America

## Abstract

**Background:**

Under the direction and sponsorship of the National Cancer Institute, we report on the first pre-clinical trial of the Comparative Oncology Trials Consortium (COTC). The COTC is a novel infrastructure to integrate cancers that naturally develop in pet dogs into the development path of new human drugs. Trials are designed to address questions challenging in conventional preclinical models and early phase human trials. Large animal spontaneous cancer models can be a valuable addition to successful studies of cancer biology and novel therapeutic drug, imaging and device development.

**Methodology/Principal Findings:**

Through this established infrastructure, the first trial of the COTC (COTC001) evaluated a targeted AAV-phage vector delivering tumor necrosis factor (RGD-A-*TNF*) to αV integrins on tumor endothelium. Trial progress and data was reviewed contemporaneously using a web-enabled electronic reporting system developed for the consortium. Dose-escalation in cohorts of 3 dogs (n = 24) determined an optimal safe dose (5×10^12^ transducing units intravenous) of RGD-A-*TNF*. This demonstrated selective targeting of tumor-associated vasculature and sparing of normal tissues assessed via serial biopsy of both tumor and normal tissue. Repetitive dosing in a cohort of 14 dogs, at the defined optimal dose, was well tolerated and led to objective tumor regression in two dogs (14%), stable disease in six (43%), and disease progression in six (43%) via Response Evaluation Criteria in Solid Tumors (RECIST).

**Conclusions/Significance:**

The first study of the COTC has demonstrated the utility and efficiency of the established infrastructure to inform the development of new cancer drugs within large animal naturally occurring cancer models. The preclinical evaluation of RGD-A-*TNF* within this network provided valuable and necessary data to complete the design of first-in-man studies.

## Introduction

The current cancer drug development path involves a linear development plan that includes assessment of efficacy in small animals and safety assessments in non-tumor bearing large animals that lead to first-in-man clinical trials. For many reasons this preclinical development process has been criticized for its inability to identify drugs that are most likely to succeed in the human clinic. For example the use of one species (i.e. rodents) to define efficacy and a second species to define safety (i.e. the dog) precludes assessment of therapeutic index until an agent actually enters early phase human studies. The biological complexity and heterogeneity of cancer is not adequately represented by the numbers of rodent tumor models commonly used in preclinical efficacy screening [1]. Complex relationships between drug exposure and necessary biological changes in tumor tissue or circulating space are not easily modeled in murine cancers. As a result, many questions are left unanswered before early phase human studies. Similar if not more pressing questions persist following early phase human trials that may significantly limit the optimal design of later phase human studies.

Spontaneous cancers in companion (pet) dogs offer a unique, and largely unexploited translational research opportunity for cancer imaging, device and drug development [2], [3]. This field of study, known as comparative oncology, has a long history of advancing surgical techniques, such as limb sparing for pediatric sarcoma patients, elucidating hyperthermia and radiobiology, and evaluating novel anti-cancer agents and delivery mechanisms, including inhalation cytokine and chemotherapy strategies [4], [5], [6], [7], [8], [9]. The features and use of cancers in pet dogs that may contribute to our understanding of cancer pathogenesis, progression and therapy have also been recently reviewed [2]. The opportunity to assess drug exposures, toxicity and efficacy (therapeutic index) in a single large animal model is highly informative. These cancers more accurately recapitulate the heterogeneity and complexity of human malignancies and as a result are linked to the problems of recurrence, resistance and metastasis. The size of the dog and tumors in these dogs make assessment of correlative endpoints such as pharmacodynamic changes in a tumor or secondary tissue quite feasible. Accordingly, these models are well suited to be integrated with existing model approaches and optimize the drug development path.

In order to take advantage of the model opportunities provided by pet dogs that have naturally developed cancer, the National Cancer Institute's Center for Cancer Research-Comparative Oncology Program (CCR-COP) has recently developed an infrastructure that is capable of multi-center nation-wide trials in tumor-bearing dogs using cancer drugs that are under development for human patients. Referred to as the Comparative Oncology Trials Consortium (COTC), this infrastructure includes 18 state-of-the-art academic veterinary oncology centers. COTC trials are centrally managed and designed to include multiple endpoints that define safety, biological and clinical activity of novel treatment agents.

The first completed trial of the COTC is an example of a pharmacodynamically focused study conducted to directly inform next step decisions in the clinical development of an adeno-associated virus phage [10], [11], [12] delivery of tumor necrosis factor-α (RGD-A-*TNF*) to αV integrins to tumor and tumor-associated vascular endothelium. The development of anti-angiogenic and vascular-targeted agents has been complex and with incommensurate results from studies in tumor-bearing mice and human cancer patients [13], [14]. It is reasonable that that some of the attributes of the comparative oncology approach may be particularly informative in the evaluation of both anti-angiogenic agents and novel gene delivery methods [15]. Accordingly, the first trial of the COTC has evaluated this ligand-direct targeting gene delivery system [10], [11], [12], [16], [17], [18]. Through a step-wise trial design we demonstrated the safety, tumor-selective trafficking, and anti-tumor activity of RGD-A-*TNF* to tumor blood vessels and targeted expression of TNFα in a relevant clinical setting. These results provide a strong pre-clinical basis for first-in-man studies in human cancer patients. In a larger context, this report of the first clinical trial from the COTC, suggests that the existing population of dogs with spontaneous tumors and this advanced clinical trial infrastructure can serve as an efficient intermediate step in the translation of new cancer treatments from pre-clinical models to man.

## Results

### Dose-escalation and single-dose safety trial phase

**Figure 1:**
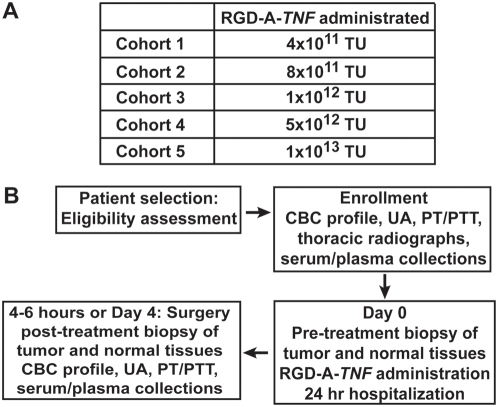
Schema representing the schedule for the dose escalation phase of RGD-A-*TNF* evaluation in dogs with spontaneous cancers. (A) This study was structured as a dose-escalation using a modified Fibonacci design to govern dose escalation towards an MTD. (B) Three dogs were enrolled in the starting-dose cohort, and three dogs per cohort were enrolled there after for each of the five dose levels planned. Dogs were scheduled to receive RGD-A-*TNF* on day zero and to undergo definitive tumor resection 4 days later. This initial 4-day group was designed (i) to evaluate vector localization and TNFα expression within tumors and (ii) to verify that the tentative follow-up schedule of RGD-A-*TNF* administration at one-week dosing intervals was biologically appropriate. After a group of dogs (n = 18) were treated according to this schedule, an additional group was enrolled by equivalent inclusion criteria to receive RGD-A-*TNF* on the same day of definitive surgical resection. In this subset of dogs (n = 6), surgery was performed 4–6 hours post administration of RGD-A-*TNF,* this same-day experimental subset was designed to establish the acute selectivity of RGD-A-*TNF* trafficking and its tumor vascular localization.

Modified Fibonacci rules of dose escalation were followed within five dose cohorts (n = 3) each receiving a single administration of RGD-A-*TNF* (Fig 1A, Table 1). All dogs underwent pre-treatment biopsy of tumor and normal tissues (Fig 1B). Dogs in the initial five cohorts (n = 18) received RGD-A-*TNF* followed by definitive surgical resection of their tumors four days later (tumor and control normal tissues were collected post treatment); an additional sixth cohort (n = 6) received RGD-A-*TNF* and underwent definitive surgery on the same day (4–6 hours following intravenous administration) (Fig 1B). At enrollment in the dose-escalation trial phase of the study, there were 9 primary bone sarcomas and 15 soft tissue sarcomas (oral cavity 3; axial or appendicular 12) (Table 1). Age (range, 2.7–14.1 years; median 9.7 years), weight (range, 15–61.2 kg; median 34.4 kg) and breed (11 mixed-breed and 13 purebred) of the dogs that entered this trial phase were recorded.

**Table 1:** RGD-A-*TNF* Dose Escalation Study: Patient Population and RGD-A-*TNF* Trafficking Data

Dose (TU)	Cohort	*Patient I.D.	Post tx. biopsy	Tumor histology	Tumor location	Age (yrs)	Weight (kg)	IF
4×10^11^	1	1.1	day 4	OCS	Oral-maxilla	9.1	25.0	+
4×10^11^	1	1.2	day 4	**PCT	Oral-maxilla	12.0	29.6	−
4×10^11^	1	1.3	day 4	**BCT	L Forelimb	14.1	32.7	+
4×10^11^	1	1.4	day 4	OS	R Proximal Humerus	7.0	30.3	−
4×10^11^	1	1.5	day 4	NST (STS)	R Forelimb	8.1	37.2	−
4×10^11^	1	1.6	day 4	STS	R Hindlimb	11.0	36.4	−
8×10^11^	2	1.7	day 4	NST (STS)	L Forelimb	12.8	32.7	−
8×10^11^	2	1.8	day 4	STS	L Hindlimb	9.9	44.0	−
8×10^11^	2	1.9	day 4	NST (STS)	R Forelimb	8.6	45.9	−
1×10^12^	3	1.10	day 4	FS	Oral-mandibular	9.4	35.0	N.A.
1×10^12^	3	1.11	day 4	NST (STS)	R Thorax	9.6	32.0	−
1×10^12^	3	1.12	day 4	STS	R Hindlimb	7.7	34.2	−
5×10^12^	4	1.13	day 4	OS	L Tibia	10.1	32.6	+
5×10^12^	4	1.14	day 4	STS	R Hip/thigh	12.5	16.3	−
5×10^12^	4	1.15	day 4	STS	Thorax	2.7	44.9	+
1×10^13^	5	1.16	day 4	OS	R Radius	4.5	41.0	−
1×10^13^	5	1.17	day 4	STS	R Thorax	10.7	40.0	+
1×10^13^	5	1.18	day 4	OS	L humerus	10.9	34.5	+
4×10^11^	1	1.19	4–6 hr	OS	R Femur	10.9	33.3	−
4×10^11^	1	1.20	4–6 hr	OS	R Tibia	11.4	31.8	+
4×10^11^	1	1.21	4–6 hr	OS	R Humerus	9.5	43.0	−
1×10^13^	5	1.22	4–6 hr	OS	L Radius	6.2	42.3	+
1×10^13^	5	1.23	4–6 hr	OS	R Radius	9.8	61.2	+
1×10^13^	5	1.24	4–6 hr	NST	L Axilla	8.1	15.0	+

TU: transducing units; IF: immunoflourescent staining for presence of RGD-A-*TNF* in tumors; OCS: osteochondrosarcoma; PCT: plasma cell tumor; BCT: basal cell tumor; NST: nerve sheath tumor; STS: soft tissue sarcoma; OS: osteosarcoma; FS: fibrosarcoma; N.A.: not analyzed due to improper shipment.

* Patient I.D.: dog is identified as participating in study 1 (dose escalation study), followed by patient number (i.e. patient 1.2 is dog 2 in study 1 and so on);

** These two tumors originally diagnosed as soft tissue sarcomas were reclassified upon histopathologic review by a single pathologist (SN).

All data were reported by contemporaneous electronic reporting such that adverse events were uniformly monitored and managed within this multi-center COTC trial design. Single-doses of RGD-A-*TNF* were for the most part well tolerated in all dose-escalation cohorts planned for this phase of the study. The only significant adverse event observed occurred during the intravenous infusion of RGD-A-*TNF* in the highest dose cohort (10^13^ TU), where a single dose-limiting toxicity (DLT) was noted (Dog #1.18; Grade 3 hypersensitivity reaction); this event (nausea, tachycardia, and hypotension) was transient and resolved with minimal supportive care. Three additional dogs were entered into this dose cohort with no further toxicity observed. No maximally tolerated dose (MTD) was reached since the highest dose failed to result in any dose limiting toxicities. There were no clinically significant neurological, cardiac, respiratory, gastrointestinal, renal, or hematologic toxicities related to the treatment of the dogs entered in this phase of the study. Moreover, there were no delays in wound healing (surgical incision) detected, or febrile episodes associated with single-doses of RGD-A-*TNF* These data suggest that RGD-A-*TNF* was safe following intravenous administration of a single dose of RGD-A-*TNF* up to 10^13^ TU. Since no MTD was achieved, it is possible that higher single doses of RGD-A-*TNF* may also be safely administered.

### Trafficking to tumor vascular endothelium and targeted TNFα delivery

**Figure 2:**
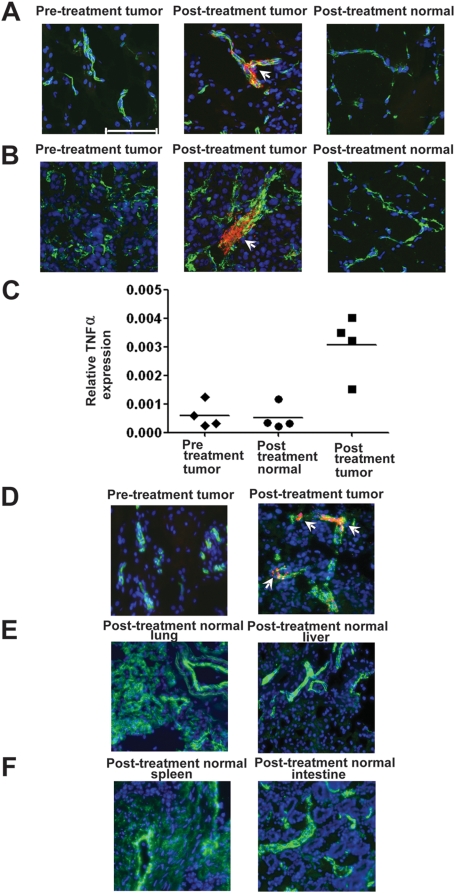
RGD-A-*TNF* trafficking resulted in selective tumor endothelial cell localization and TNF *α* expression. (A, B) RGD-A-*TNF* selectively targeted tumor-associated vasculature (arrows) and was absent from normal tissues at 4–6 hours (A) and at 4 days (B). Magnification, 400-fold; scale bar, 100 µM. Pre-treatment tumor biopsies, post-treatment tumor biopsies, and post-treatment normal tissues in dogs that received a single-dose of RGD-A-*TNF* double-stained with an anti-CD31 specific antibody plus an anti-bacteriophage specific antibody. Detection was performed with Alexa Fluor 488 (green, blood vessels), Alexa Fluor 594 (red, AAVP), and DAPI (blue, cell nuclei). (C) Pre-treatment tumor biopsies (day 0), post-treatment normal biopsies (day 4) and post-treatment tumor biopsies (day 4) were used for extraction of total RNA. RT-PCR was performed to measure transcript levels of human TNFα in quadruplicate. The Y-axis represents the relative TNFα expression levels in post-treatment normal biopsies and post-treatment tumor biopsies compared to pre-treatment tumor biopsies after normalization to GAPDH expression (Kruskal-Wallis Test, p = 0.0107). All data are presented as means±standard deviations. (D–F) Presence of RGD-A-*TNF* was evaluated in post-treatment (day 28) necropsy samples of tumor (D) and normal tissues (E, F). Tissues were stained for RGD-A-*TNF* as described earlier. RGD-A-*TNF* selectively targeted tumor-associated vasculature in post-treatment tumor samples (arrows). In contrast, the vector was not apparent in pre-treatment tumor samples or in post-treatment normal control necropsy samples (such as lung, liver, spleen or intestine) after serial administrations of RGD-A-*TNF*.

To determine if RGD-A-*TNF* trafficked to tumor versus normal tissues, we analyzed pre-treatment and post-treatment (after 4–6 hours and after 4 days) biopsies of tumor and normal tissues (i.e., oral mucosa, skin, and muscle) following a single dose of RGD-A-*TNF* by dual-color immunofluorescence (IF) staining for phage (Fig. 2A, B). The staining pattern of all biopsy samples were scored as positive or negative as described (Methods). Clear co-localization of RGD-A-*TNF* particles were seen only within tumor vascular endothelium in tumor biopsies, but not in the blood vessels of normal tissues, as early as 4 to 6 hours post-treatment (Fig. 2A) and at 4 days post-treatment (Fig. 2B). The specificity of the IF assay was supported by the lack of staining seen in the pre-treatment biopsies in any dogs (Fig. 2A, B). Secondarily, there was no tumor cell RGD-A-*TNF* staining evident, only tumor vascular endothelium staining. We observed heterogeneous RGD-A-*TNF* staining patterns in different sections from the same tumor biopsy and in different biopsies within the same tumors. This reflects the heterogeneity of tumor vasculature biology in spontaneous cancer that led to differential uptake of RGD-A-*TNF* in tumor tissues. Consistent trafficking of RGD-A-*TNF* to tumor blood vessels was observed in the two cohorts receiving the highest doses; specifically, two out of three dogs (67%) in the cohort that received 5×10^12^ TU and five out of six dogs (83%) in the cohort that received 1×10^13^ TU. There was no apparent association between tumor histology and tumor vascular targeting.

After establishing that RGD-A-*TNF* preferentially localizes within tumor vascular endothelium after systemic administration, we next evaluated whether vector localization would result in targeted TNFα gene expression. Three out of the four dogs tested for hTNFα gene expression showed significantly increased levels of hTNFα in post-treatment tumor biopsies (day 4) compared to pre-treatment tumor biopsies (Kruskal-Wallis test p = 0.0107) after a single dose of RGD-A-*TNF* (Fig. 2C). In contrast, post-treatment biopsies of normal tissues showed no detectable increase in levels of hTNFα compared to pre-treatment tumor biopsies (Kruskal-Wallis test, p>0.05).

### Multi-dose phase of study

**Figure 3:**
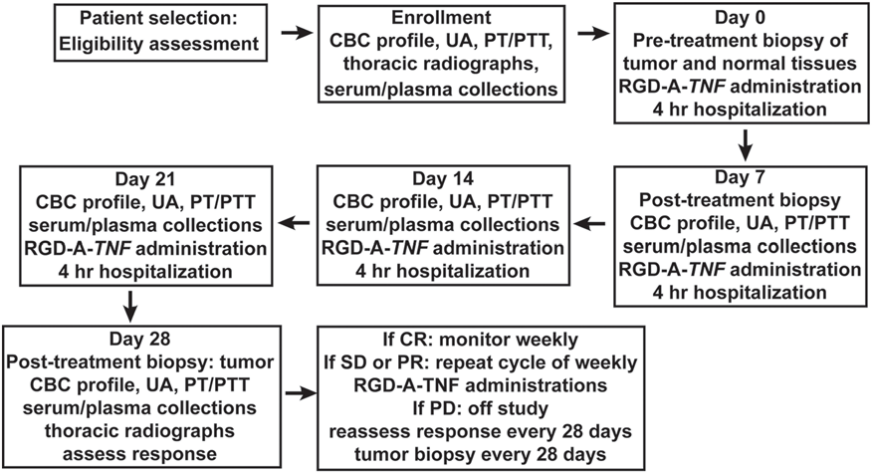
Schema representing the schedule for the multi-dose phase of RGD-A-*TNF* evaluation in dogs with spontaneous cancers. This study was designed as an open label, multiple fixed-dose trial (i) to establish feasibility and (ii) to identify chronic and/or cumulative toxicity of repetitively administered RGD-A-*TNF*. Dogs received weekly doses (5×10^12^ TU intravenously) of RGD-A-*TNF*. Anticancer activity of this agent was evaluated using RECIST criteria. The treatment received population included dogs that received at least four weekly doses (i.e., one cycle 1). This population consisted of 14 dogs. Dogs were permitted to receive additional therapy in subsequent cycles if there was evidence of either stable disease or tumor response. Tumor measurements were recorded every two weeks with full restaging every 28 days.

In the multi-dose phase of the study, a cohort of dogs (n = 18) was enrolled and serially treated with fixed systemic doses (5×10^12^ TU per week) of RGD-A-*TNF* (Fig 3). Tumor biopsies were obtained pre-treatment (deemed day zero) and on days 7, 28 and 56 post-treatment (Fig 3). At enrollment in the multi-dose phase of the study, pathological tumor types (Table 2) included soft tissue sarcomas (n = 8), malignant melanomas (n = 5), osteosarcoma (n = 1), multilobular osteochondrosarcoma (n = 1), hemangiosarcoma (n = 1), lymphoma (n = 1), and squamous cell carcinoma (n = 1). All tumors were located at peripheral sites that were accessible to biopsy; however, concurrent metastatic disease existed in two dogs. Age (range, 6–14.8 years; median 10.2 years), weight (range, 11–55 kg; median 33.3 kg) and breed (7 mixed-breed and 11 purebred) of the dogs that entered this trial phase were recorded (Table 2).

**Table 2:** RGD-A-*TNF* Multiple Dose Study: Patient Data and Tumor Responses

*Patient I.D.	Tumor histology	Age (yrs)	Cycle 1 tumor measurements day0/day14/day28 (cm)	Cycle 1 Response (% change)	Cycle 2 tumor measurements day28/day42/day56 (cm)	Cycle 2 Response (% change)
2.1	FS	11	5.0/7.0/9.5	PD (90% inc.)		
2.2	Sarcoma	10.2	23.0/21.0/19.0	SD (18% dec.)		
2.3	Melanoma	12.8	2.0/2.0/2.0	SD (0%)		
2.4	Sarcoma	14.8	14.7	Unevaluable		
2.5	Sarcoma	10.4	6.8/8.5/8.2	PD (21% inc.)		
2.6	OS	9	3.2/4.5/7.4	PD (130% inc.)		
2.7	Lymphoma	8.6	11.6/14.2	SD	14.2/15.8	PD (11% inc, uveitis)
2.8	Hemangiosarcoma	7.1	6.5/8.2/6.5	SD (0%)	6.5/7.1/6.5	PD (pulm mets)
2.9	PNST	10.3	5.6	Unevaluable		
2.10	SCC	12.8	4.6	Unevaluable		
2.11	S-C MS	9.9	12.3/7.0/8.2	PR (33% dec.)	8.0/2.9/1.85	PR (85% dec.)
2.12	PNST	8.8	4.0/4.2/4.5	SD (12% inc.)		
2.13	PNST	12.1	6.6/8.0/8.1	PD (22% inc.)		
2.14	Melanoma	11	3.9/4.6/6.0	PD (54% inc.)		
2.15	MLT	6.3	3.5/3.5/3.5	SD (0%)		
2.16	Mel – LN mets	10	3.5/3.0/2.6	SD (26% dec.)	2.6/2.2/2.3	PR (33% dec.)
2.17	Melanoma	11	9.0/5.5/5.8	Unevaluable		
2.18	Melanoma	6	14.6/23.2	PD (59% inc.)		

FS: fibrosarcoma; OS: osteosarcoma; PNST: peripheral nerve sheath tumor (malignant); SCC: squamous cell carcinoma; S-C MS: subcutaneous myxosarcoma; MLT: multi-lobular tumor; Mel: LN mets: melanoma lymph node metastases; inc.: increase; dec.: decrease; PD: progressive disease; SD: stable disease (shown in yellow); PR: partial response (shown in green); Unevaluable: due to consent withdrawal by the owner, or death or euthanasia before completion of cycle 1

* Patient I.D.: dog is identified as participating in study 2 (fixed multiple dose study), followed by patient number (i.e. patient 2.2 is dog 2 in study 2 and so on)

Cycle 1: dogs received 4 weekly doses of RGD-A-*TNF*; Cycle 2: dogs received additional 4 weekly doses of RGD-A-*TNF*

Serial, multiple fixed doses (total, 78.25 doses; mean 4.34 doses/dog; range, 1–8 doses/dog) were administered. Fourteen dogs in this cohort (78%) completed at least one full cycle (defined as four weekly doses) of treatment and were included in the treatment-received population for assessment of tumor response. Death due to disease progression (n = 2) or study withdrawal by owner request (n = 2) was the most common reason for dogs to fail completion of treatment cycle 1. Three of 14 dogs (21%) completed a second full cycle (i.e., eight weekly doses) of treatment.

Similar to the dose-escalation phase of the study, all data was electronically reported within the format of this COTC multi-center trial design. Furthermore, the same clinical evaluation, imaging, and laboratory studies were obtained before and after the administration of each weekly dose of RGD-A-*TNF.* In dogs that received repetitive weekly doses, the most common toxicity (n = 9) was a grade 3 hypersensitivity reaction during intravenous administration; six dogs out of 14 (42%) had more than one clinical episode (i.e., vomiting, hypotension, and tachycardia) that was not prevented by pre-medication (famotidine, diphenhydramine and dexamethasone). However, all of these adverse events were transient and resolved following temporary pause (<30 min) of administration and minimal supportive care, after which a slower intravenous infusion rate (100 ml/hour) was resumed without further complications. There were no apparent long-term clinical sequelae to these events. Grade 3 or 4 non-neutropenic fever was noted in five dogs (36%) in the study (three noted during the infusion and two on non-administration days). All fever episodes resolved with symptomatic care. One case (Dog #2.2) presented with grade 3 necrosis (open wound and drainage at the tumor site) on treatment day 19; this same dog had an 18% reduction in tumor size at that time (defined by RECIST as stable disease). There were three other events with unknown attribution, including a grade 2 skin reaction associated with demodectic mange (Demodex *canis*), a grade 1 ventricular arrhythmia, and a first-event-death of unknown cause two days after the fifth dose of RGD-A-*TNF*. In the first-event-death case, no adverse events were noted (Dog #2.14) during the week prior to this event. First-event-death in dogs with advanced cancer (not unlike early phase human trials) is not uncommon and may be associated with disease progression, particularly when observed as isolated events. No other significant adverse events including clinical or laboratory reports of neurological, cardiac, respiratory, gastrointestinal, renal, or hematologic toxicity related to serial treatment with RGD-A-*TNF* were seen. Three dogs out of 14 (21%) were euthanized due to progressive disease and underwent rapid (warm) necropsy; pathologic evaluation of visceral organs (brain, heart, lung, liver, spleen, gastrointestinal tract, kidneys, and lymph nodes) did not detect any treatment related histologic abnormalities in these organs; data supporting the relative safety and tumor-targeting of RGD-A-*TNF*. In select warm necropsy cases (n = 2), two-color IF was performed and tumor-specific trafficking of RGD-A-*TNF* was seen (Fig. 2D); no RGD-A-*TNF* was detected in examined normal tissues (lung, liver, spleen, and gastrointestinal tract) (Fig. 2E, F).

### Treatment-associated objective response

**Figure 4:**
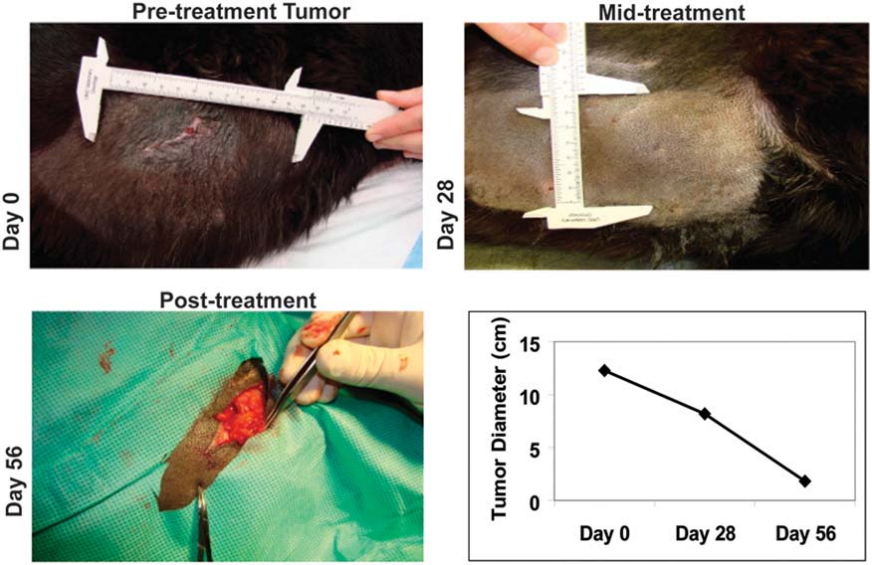
RGD-A-*TNF* administration resulted in objective tumor responses in dogs with spontaneous cancers. A large primary soft tissue sarcoma on the flank of Dog #2.11 is shown to feature the potential magnitude of the tumor response. Prior to therapy (day 0), the tumor measured 12.3 cm in longest diameter. At day 28, after 4 weekly infusions of RGD-A-*TNF*, the tumor measured 8.2 cm in longest diameter (a 33% regression) and a RECIST-based partial response (PR). At day 56, after a total of 8 weekly systemic infusions of RGD-A-*TNF*, the tumor measured 1.85 cm in longest diameter prior to resection. Therefore, this response equated to an 85% regression from baseline and a continued clinical PR. Upon surgical resection of the residual lesion, no viable tumor was found and a pathological complete response was determined.

In the treatment-received population of 14 dogs, we used serial tumor measurements to determine the activity of RGD-A-*TNF* according to the Response Evaluation Criteria in Solid Tumors (RECIST) (Table 2). Again, the four dogs that failed to complete one cycle (4 weeks) of therapy were excluded for evaluation of response. Evaluable dogs included those with tumors not amenable to surgical excision, tumors for which prior therapy had failed, and tumors in which standard-of-care therapy was declined. Two animals (Dogs #2.11 and #2.16) had a RECIST-defined partial response (PR) (14%; 95% confidence interval 2–43%) one at day 28 and the other at day 56 (Table 2). Six dogs had stable disease (SD) (43%; 95% confidence interval 18–71%) including a dog with an 18% reduction in tumor size, and six dogs had disease progression (PD) (43%; 95% confidence interval 18–71%) after one cycle of therapy. After two cycles of therapy, two of the dogs with previously defined stable disease at Day 28 progressed (Table 2). The case of a marked clinical PR is depicted to illustrate the magnitude of the serial therapeutic effect of RGD-A-*TNF* in a dog presenting with a large soft tissue sarcoma (Fig. 4). This dog (#2.11) had achieved an ≥85% reduction in what had previously been unresectable disease. Following this response a <2 cm residual lesion was resected resulting in a pathological review revealing no viable tumor. These results support the previous studies evaluating the safety and activity of this strategy in tumor-bearing rodent models [10], [19].

### RGD-A-*TNF* directed immune response in treated dogs

We analyzed pre-treatment and post-treatment serum samples from single-dose and serial multi-dose study dogs to measure anti-AAVP antibodies. Collectively, we observed 1.8 to 2.0 fold increases in anti-AAVP antibody titers post treatment when compared to pre-treatment levels. However, we did not observe further increases in anti-AAVP antibodies over time in the serial multi-dose treated dogs (Day 7 vs. Day 28 vs. Day 56).

## Discussion

The opportunity to integrate cancers that naturally develop in pet dogs within the development path of a novel human cancer drug is further realized through the inaugural study of the COTC. In this report, we describe a targeted delivery of TNFα with an AAVP gene delivery system to tumor blood vessels of pet dogs with spontaneous cancer. Selective AAVP homing, tumor-associated vascular expression of TNFα, systemic safety, and RECIST-based objective responses were observed (same species therapeutic index). These large animal spontaneous cancer models are well suited to inform pre-clinical to clinical transitions necessary for successful drug development and compliment the use of both existing rodent models and human clinical trials.

The COTC is an active network of academic comparative oncology centers, centrally managed by the NCI's Comparative Oncology Program. The COTC designs and implements clinical trials in dogs with cancer with the goal of providing necessary translational data for novel therapies, techniques or devices for future cancer patients. Trials are executed at COTC member institutions, which currently include eighteen veterinary teaching hospitals across the United States. As described above, trials conducted by the COTC may include several biological and clinical endpoints that can be directly integrated into the design of human Phase I and II clinical trials. Although not included in the study presented here, correlative imaging strategies, such as PET/CT or dynamic MRI, with the ability to link these imaging endpoints to tumor or circulating biological surrogates are feasible through the designed infrastructure. Such complex correlative studies may be vetted in a clinically relevant setting through COTC studies, and therein provide a model to evaluate all parts of this process including tissue collection standards and techniques, timing of imaging and biopsy, and assay methodologies prior to early phase human studies.

In the current study, COTC001, we assessed the fluidity and structure of this novel preclinical infrastructure. In the process, we defined the safety and efficacy of RGD-A-*TNF* targeting to tumor endothelium in pet dogs with spontaneous cancer. RGD targeted delivery to tumor αV integrins has been previously described [20], [21] and this anticipated targeting was the basis of the RGD-A-*TNF* vector development [11], [19], [22]. In previously published work in mouse xenograft models, we described targeted systemic delivery of RGD-A*VVP* vectors expressing either HSVtk or TNF-α, to tumor vasculature [12], [22], [23], [24]. Anti-tumor activity has been seen in Kaposi sarcoma, bladder carcinoma, prostate carcinoma and melanoma [12], [22]. Although, activity of RGD-A-*TNF* has been demonstrated in traditional small animal models, the comparative oncology approach provided unique information regarding the safety of RGD-A-*TNF* that would not have been possible in conventional animal safety studies. Since neither tumor nor tumor vasculature are present in healthy animals (i.e. purpose-bred research dogs), a safety assessment in these animals would likely under-report adverse events related to RGD-A-*TNF.* Drug-related events reported in our population of tumor-bearing pet dogs were indeed generally mild and self-limiting. In fact, a MTD was not achieved in the single dose cohorts evaluated in the dose-escalation as increasing the dose of RGD-A-*TNF* was limited by manufacture process. Most adverse events that were documented were seen either during the administration or within 2 hours of administration in dogs that had received multiple doses of RGD-A-*TNF.* Administration-related events included hypersensitivity-like reactions and fever. Given the per-acute nature of these events, it is plausible that such they were related either to undetectable remnant endotoxin in the treatment product or to a specific response to the vector itself. Testing of all production lots of RGD-A-*TNF* failed to reveal residual endotoxin; however, one cannot entirely exclude this or other contaminants as a cause of some of the adverse events.

Serial tumor and control tissue biopsies taken before and after the administration of RGD-A-*TNF* provided an opportunity to correlate drug exposure with tumor and normal tissue trafficking of RGD-A-*TNF.* These experiments validated the tumor-specific targeting of the RGD-A-*TNF* in the setting of dogs with spontaneous cancers. We observed vector localization in tumor vascular endothelium in post-treatment tumor biopsies taken 4–6 hours and 4 days after systemic administration of RGD-A-*TNF.* Notably, there was a complete absence of the vector in normal tissue biopsies in all treated dogs. Thus, RGD-A-*TNF* exploits aberrant disease-related vasculature to target the therapy of interest specifically to the tumor. Thus confirming the safety of this delivery system first observed in our small animal models [22]. Consistent with this, warm necropsies from dogs euthanized due to disease progression showed that RGD-A-*TNF* targeted tumor vasculature but not blood vessels within normal visceral organs. Importantly, we did not observe RGD-A-*TNF* in any of the control tissues analyzed, including liver whereas the presence of RGD-A-*TNF* has been previously seen in the liver and spleen of rodents treated with RGD-A-*TNF* [22]. These large animal data are particularly valuable as the risks and benefits for AAVP delivery strategies in human cancer patients are considered. Effective targeting of RGD-A-*TNF* was seen at doses from 5×10^12^ to 10^13^ TU. A dose of 5×10^12^ TU was selected as optimal dose for the multiple-dose study, due to equivalent trafficking, targeting, and safety as well as our inability to produce 1×10^13^ TU in a timely manner. At this dose vector targeting also resulted in measurable expression of human TNFα. Further support for the biological relevance of the observed trafficking of RGD-A-*TNF* along with targeted expression of human TNFα, was provided by the objective anti-tumor activity observed in dogs receiving multiple weekly treatments. RECIST-based responses were observed in two dogs with soft tissue sarcoma and metastatic melanoma; such objective tumor responses are particularly germane, because all dogs in this study had large bulky tumors and were not candidates for conventional loco-regional treatments such as surgery or radiation therapy. We speculate that the observed objective responses were the result of TNFα transgene expression, as no activity was seen in our mouse models from either non-targeted AAVP or targeted-null vector [22]. The proposed mechanism for this activity is the induction of endothelial cell apoptosis and hyperpermeability leading to hemorrhagic necrosis in treated tumors [25], [26], [27]. This biology will be further explored in planned canine studies using Good Manufacturing Practice (GMP) quality RGD-A-*TNF,* via caspase-3 and CD31 staining. However, it is important to note that the future development strategy for this delivery system is not as a single agent. Instead it will likely involve combinational therapies, either dual transgene insertion or adjunct administration of complementary agents. Hence its single agent activity is note worthy.

In summary, the biological complexity of naturally occurring cancers in pet dogs, their size and strong similarities to human cancers, and the availability of a motivated population of pet-owners interested in treatments for their pets with cancer provide an opportunity to now develop a comparative and integrated approach to cancer drug development. [1], [2], [3]. The first study of the COTC (COTC001) provides an example of this integration and a functional infrastructure that may deliver trial outcomes in a timely manner. Specifically, COTC001 assessed the safety, selective tumor-specific localization, and anti-tumor activity of RGD-A-*TNF* in dogs with measurable malignant cancers. This report supports a new paradigm for rapid intermediate evaluation of agents prior to or after early human trials as a means to creating a more informed and optimal cancer drug development pathway.

## Materials and Methods

### Comparative Oncology Trials Consortium

The over-riding goals and infrastructure of the COTC have been recently described [2], [3]. This report represents the first clinical trial in dogs with cancer through this multi-institutional consortium. All COTC trial data was reported electronically and contemporaneously reviewed through a modified form of Oracle Clinical, known as the Cancer Central Clinical Database (C3D), developed through the NCI's CCR and Cancer Bioinformatics Grid (CaBIG), modified for use in canine clinical trials [28].

### Trial eligibility and enrollment

Client-owned pet dogs with biopsy-proven malignant tumors (newly-diagnosed or recurrent disease) with favorable performance status (Grade 0 or 1 Modified ECOG Performance Status), and informed owner consent were eligible for enrollment. For the dose-escalation phase, entry criterion was an externally measurable tumor (>2 cm in the longest diameter) that was amenable to surgical resection. For the multiple-dose trial phase entry criteria included measurable tumors of greater than 3 cm in the longest diameter. Physical examination, laboratory studies, and imaging studies were performed to evaluate eligibility prior to enrollment (Figs 1 & 3). Specifically, complete blood count, biochemical screening profile, urine analysis, prothrombin time and partial thromboplastin time, and a baseline electrocardiogram were required. Exclusion criteria removed dogs weighing less than 15 kg, those with significant co-morbidities (such as renal, liver, and heart failure or coagulopathy) a diagnosis of mast cell cancer, or concurrent chemotherapy, radiation therapy, or biological therapy. Tumor staging included thoracic radiographs and abdominal ultrasound when clinically indicated. Dogs with metastatic disease were excluded from entry in the dose-escalation phase (Table 1) but were allowed in the multiple-dosing phase of the trial (Table 2). All dogs were evaluated uniformly and treated in a defined clinical protocol with Institutional Animal Care and Use Committee (IACUC) approval at each COTC enrollment site (Colorado State University, University of Missouri, University of Pennsylvania, University of Tennessee, and University of Wisconsin). The NCI-COP reviewed the eligibility screening and approved trial entry of each individual dog.

### Dose-escalation phase

This study was structured as a dose-escalation using a modified Fibonacci design to govern dose escalation towards a MTD (Fig 1). Three dogs were enrolled in the starting-dose cohort, and three dogs per cohort were enrolled thereafter for each of the five dose levels planned (Fig 1A, Table 1). Dogs were scheduled to receive RGD-A-*TNF* on day zero and to undergo definitive tumor resection 4 days later (Fig 1B). This initial 4-day group was designed (i) to evaluate vector localization and TNFα expression within tumors and (ii) to verify that the tentative follow-up schedule of RGD-A-*TNF* administration at one-week dosing intervals was biologically appropriate. After a group of dogs (n = 18) were treated according to this schedule, an additional group was enrolled by equivalent inclusion criteria to receive RGD-A-*TNF* on the same day of definitive surgical resection (Fig 1B). In this subset of dogs (n = 6), surgery was performed 4–6 hours post administration of RGD-A-*TNF,* this same-day experimental subset was designed to establish the acute selectivity of RGD-A-*TNF* trafficking and its tumor vascular localization. After surgery, dogs returned to their participating institution for suture removal and surgical wound re-evaluation at day 14 postoperatively. A standard physical examination was performed at that time.

### Multi-dose trial

This study was designed as an open label, multiple fixed-dose trial (i) to establish feasibility and (ii) to identify chronic and/or cumulative toxicity of repetitively administered RGD-A-*TNF* (Fig 3). Dogs received weekly doses (5×10^12^ TU intravenously) of RGD-A-*TNF*. Anticancer activity of this agent was evaluated using RECIST criteria. The treatment received population included dogs that received at least four weekly doses (i.e., one cycle 1). This population consisted of 14 dogs. Dogs were permitted to receive additional therapy in subsequent cycles if there was evidence of either stable disease or tumor response. A standard physical exam was performed at each visit and tumor measurements were recorded every two weeks with full restaging every 28 days (Fig 3).

### Construction and production of RGD-A-*TNF*

The general design and construction of the AAVP particle has been described [10], [11], [12], [29]. An AAVP construct expressing human TNFα was created by ligation of a 880-bp NotI/HindIII fragment from pG1Si*TNFα* [30] into the vector pAAV-eGFP/NotI/HindIII, with replacement of the *GFP* gene sequence. Subsequently, AAV-*TNFα* containing inverted terminal repeats (ITRs) was ligated into the RGD-4C AAVP/PvuII to obtain the RGD-A-*TNF* vectors. RGD-A-*TNF* is a targeted vector with binding affinity to αV integrins.

To obtain targeted AAVP particles, RGD-A-*TNF* was electroporated into MC1061 *E. coli,* and virus particles purified from culture supernate as described [10], [11], [12]. Contaminating bacterial cells were removed by repeated centrifugation at 10,000 rpm for 10 min. The RGD-A-*TNF* prepared from MC1061 *E. coli* was used to purify large-scale AAVP particles from a permissive host bacterial strain (k91Kan *E. coli*). After preparation of RGD-A-*TNF*, endotoxin was removed using MiraCLEAN endotoxin removal kits (Mirus Bio Corp., Madison, Wisconsin) with pyrogen-free materials and under good laboratory practice (GLP) conditions in a sterile hood equipped with a high efficiency particulate air (HEPA) filter. Briefly, 0.1 volumes of MiraCLEAN buffer were added to RGD-A-*TNF* preparation, followed by vortexing and incubation on ice for 15 min. After incubation, 0.03 volumes of the EndoGO extraction reagent were added and the solution was incubated on ice for 15 min with intermittent vortexing. Samples were incubated at 50°C for 15 min. Phases were then separated by centrifugation at 13,000 rpm for 2 min, and the upper colorless aqueous phase was transferred to a new tube. Every batch of RGD-A-*TNF* was underwent endotoxin removal (3 cycles). Next, AAVP was tested to determine endotoxin levels with Limulus Amebocyte Lysate (LAL) QCL-1000 kits (Cambrex, Walkersville, Maryland). All the materials used in the assay were validated endotoxin-free. Consistency among reagent batches was confirmed by standard infection of human M21 melanoma cells and measurement of TNFα in the supernatant [22]. For final quality control, RGD-A-*TNF* preparations were required to have endotoxin levels of less than 1.0 EU/ml prior to administration.

Following endotoxin removal, the final preparation was filtered through a sterile filter. To determine the number of bacterial transducing units (TU), the permissive host k91Kan *E. coli* was infected with serial dilutions of AAVP particles and plated on Luria-Bertani agar plates containing tetracycline and kanamycin. TU were determined by counts of the number of bacterial colonies and were expressed either as TU/µl or as Relative TU.

### Product packaging, shipping, and quality control

Each batch of RGD-A-*TNF* underwent determination of TNFα titer and protein expression. To assess TNFα expression, we infected human M21 melanoma cells with 10^5^ TU/cell for 3 hours at 37°C in serum-free RPMI medium. After infection, 10% serum was added to each well, and incubation was continued. The medium was replaced 72 hours later. On day 5, the culture supernate was collected for measurement of secreted human TNFα levels by ELISA (Invitrogen, Carlsbad, California).

Packaging was done into pyrogen-free sterile vials (Allergy Labs, Oklahoma City, Oklahoma) in a sterile hood equipped with a HEPA filter. RGD-A-*TNF* was diluted in sterile normal saline (Quality Biologicals Inc., Gaithersburg, Maryland) to a final volume of 5 ml. Each vial containing the vector RGD-A-*TNF* was labeled individually with batch and vial numbers, preparation and expiration dates, and viral concentration. Whenever a dog was enrolled in the study with confirmed eligibility, vials were shipped on ice overnight to the COTC-participating institution, where they were stored at 4°C until administration. Storage times at COTC institutions were less than 48 hours.

### RGD-A-*TNF* administration, monitoring, and safety assessment

Dogs underwent a complete physical examination, baseline imaging, blood tests and appropriate biopsies prior to their receiving RGD-A-*TNF* intravenously. Vital signs (core temperature, pulse, respiratory rate, arterial blood pressure), and EKG were recorded at baseline. Dogs received the pre-determined dose of RGD-A-*TNF* suspended in 100 ml of normal saline as continuous rate infusion (CRI) over 30 min. Vital signs and EKG recordings were collected every 15 min during intravenous infusion and hourly for four hours following administration of RGD-A-*TNF.* After this immediate post-treatment period, 24-hour monitoring was required for all dogs with vital signs/EKG recorded every 4 hours.

Definition of acute and chronic toxicities of single and multiple doses of RGD-A-*TNF* was a major goal of the study. Blood samples were collected to define hematologic and biochemical DLT. CBC, screening biochemical profile, urinalysis, prothrombin time, and partial thromboplastin time were evaluated at trial enrollment, pre-operatively in the dose escalation and prior to each weekly treatment in the multi-dose study. The Veterinary Cooperative Oncology Group Common Toxicity Criteria for Adverse Events (VCOG-CTCAE) was used to determine DLT [31], defined as any grade 3 or grade 4 (hematologic or non-hematologic) toxicity. MTD was defined as one dose level below the maximum achieved in dose-escalation or by chronic toxicities in the multi-dose trial phase of the study. Any and all adverse events were collected within an electronic database reporting system (C3D) [32] that followed strict reporting timelines.

### Tissue collection

In the dose-escalation phase of the study, serial biopsies were required from all dogs to determine the localization of RGD-A-*TNF* in tumors and normal tissues. Incisional pre-treatment biopsies yielded tissue samples that were immediately frozen in liquid nitrogen and subsequently fixed in 10% formalin. Excisional post-treatment tumors were acquired surgically through standard operative techniques. At surgery, at least three sections of each tumor were sampled at different angles/planes; these samples were placed separately in 10% formalin, liquid nitrogen and RNAlater (Ambion, Austin, Texas). Three sections of normal tissue from a site distant to the tumor were also sampled in a similar fashion. If possible, intra-operative “in-field” normal tissues were also collected. Finally, in the multi-dose phase of the study, pre-treatment tumor biopsies were collected (as described above for dose-escalation) and serial post-treatment tissue samples acquired on days 7, 28 and 56 of treatment if applicable.

### Immunofluorescence

To detect the presence of RGD-A-*TNF,* we stained 5 µM frozen tissue sections for two-color IF. Briefly, sections were fixed in PBS containing 1% paraformaldehyde for 10 min, followed by 2 washes in PBS for 10 min. The tissue was rendered permeable in ethanol: acetic acid (2∶1, vol∶vol) at −20°C for 5 min, incubated with Image-iT FX signal enhancer for 30 min at room temperature (RT), and washed in PBS. Non-specific binding was blocked with PBS containing 5% goat serum for 30 min at RT. The primary antibodies were applied overnight at 4°C as follows: a 1∶1,000 dilution of anti-fd bacteriophage antibody (Sigma, St. Louis, Missouri) and a 1∶20 dilution of anti-human CD31 (Dako Cytomation, Denmark). Sections were washed thrice in PBS for 5 min and were incubated with the secondary antibodies (Invitrogen, Carlsbad, California) as follows: a 1∶400 dilution of goat anti-rabbit Alexa Fluor 594 and goat anti-mouse Alexa Fluor 488, for 30 min in the dark. Finally, sections were washed four times in PBS for 2 min each, mounted in Vectashield mounting medium with DAPI (Vector Labs, Burlingame, California), and images were viewed and captured on a confocal fluorescent microscope (Zeiss LSM 510, Zeiss Inc., Germany). For each sample a minimum of 5 sections were stained and analyzed. For samples where no staining was observed, an additional 10 sections were stained and analyzed.

### RGD-A-*TNF* trafficking

“Positive” RGD-A-*TNF* IF co-localization was determined if a tumor or normal tissue sample had evident RGD-A-*TNF* and endothelial cell staining in serial biopsies from a given time point. “Negative” staining was determined if no co-localization was evident in at least 15 sections of serial biopsies from a given time point. Prior to the study's initiation, the optimal “trafficking” dose of RGD-A-*TNF* was defined in the study protocol as that which resulted in vector localization in tumor vasculature but not in the vasculature of normal tissues in at least two out of three dogs of within a dose cohort.

### Tumor response assessment

Standardized serial measurements of tumors were made prior to trial entry and weekly thereafter. The greatest diameter of each tumor was measured with calipers and was recorded (in cm) in the database. Target lesions were selected at baseline (pre-treatment) by physical examination or imaging and used for response assessment; non-target lesions were similarly identified, however, serial measurements of these lesions were not required. To determine objective responses to treatment with RGD-A-*TNF* we used RECIST criteria [12], [33], [34], [35], [36], [37]. CR was defined as disappearance of all lesions without any new lesion development; PR, a decrease by 30% or more of the longest diameter or sum of the greatest diameters of all measured target lesions; SD, any change in tumor size that did not satisfied PR or PD criteria; PD, an increase >20% in the greatest diameter or sum of the greatest diameters of all measured target lesions or any new lesions.

### TNFα expression

TNFα mRNA was assessed by RT-PCR with primer-probe sequences unique to human TNFα inserted into RGD-A-*TNF.* Total RNA was isolated from frozen tissues with Trizol (Invitrogen Corp.) and RNeasy total RNA kit (Qiagen), either in the presence or absence of DNaseI. First-strand cDNAs were generated from the total RNA either in the presence or absence of reverse transcriptase (Invitrogen Corp.). Quantitative RT-PCR was performed with a Gene Amp 7500 Sequence Detector (Applied Biosystems). Amounts of PCR products were measured as fluorescent signal intensity after standardization with a GAPDH internal control. The following sense (S) and antisense (AS) primers and probes were designed by the use of Primer Express 2.0 software (Applied Biosystems) for real time RT-PCR analysis:

S 5′ TTCAGCTCTGCATCGTTTTG 3′
AS 5′CTCAGCTTGAGGGTTTGCTACA 3′
Probe 5′ FAM-TTCTCTTGGCGTCAGATCATCTTCTCGAAC-TAMARA 3′

### Detection of AAVP antibodies

Presence of canine anti-bacteriophage antibodies was detected using ELISA techniques. Briefly, 1×10^10^ RGD-A-*TNF* particles in 50 µl PBS were coated in 96 well plates and allowed to adhere overnight at 4°C. Non-adherent particles were removed by washing with PBS followed by blocking with 5% BSA in PBS for 1 hr. Serially diluted bacteriophage anti-fd antibody (positive control) (Sigma, St. Louis, Missouri) or 100 µl of serum samples were added for 2 hr. Wells were washed seven times with wash buffer (1% BSA in PBS). The binding was detected using 1∶3000 dilution of Protein G separose (Pierce, Rockford, Illinois) for 1 hr, followed by 30 min incubation with TMB substrate (Pierce, Rockford, Illinois). Reaction was stopped by addition of 50 µl stop solution. The color reaction was read at 450 nm using BioRAD ELISA plate reader. A standard curve was generated by plotting optical density readings obtained for different serial dilutions of anti-fd antibody and the antibody titer for unknown samples were quantified.

### Necropsy and expert pathology review

Necropsies were required for all dogs on study. Dogs that were euthanized due to progressive disease received a full warm post-mortem examination when possible. Tissues were collected within 20 min and all tumor samples (primary and metastatic) were frozen in liquid nitrogen and subsequently fixed in 10% formalin. Control (normal-appearing) visceral organs (liver, spleen, heart, kidneys, lung, gastrointestinal tract, brain, and lymph nodes) were also collected, frozen, and fixed. One dog that died at home received a cold necropsy. A single veterinary pathologist (SN) reviewed all tissue samples for integrity and histopathologic evaluation.
